# Evaluation of two fixation techniques for direct observation of biofilm formation of *Bacillus subtilis in situ*, on Congo red agar, using scanning electron microscopy

**DOI:** 10.14202/vetworld.2020.1133-1137

**Published:** 2020-06-18

**Authors:** Nadia Mahmoud Tawfiq Jebril

**Affiliations:** Department of Biological Sciences, College of Sciences for Women, University of Babylon, Iraq

**Keywords:** alcian blue, *Bacillus subtilis*, biofilm, Congo red agar, scanning electron microscopy

## Abstract

**Background and Aim:**

Direct observation, scanning electron microscopy (SEM) is a common method used for the observations of biofilms. *N-*(*3*-dimethylaminopropyl)-*N’*-ethylcarbodiimide) (EDC) fixation method has proven to be a valuable fixation method in the observation of these biofilms. Still, it entails a method of biofilm fixation that can damage slim structures, leading to the impossible observation of biofilm development. In contrast, alcian blue and lysine (ABL) fixation technique appears more glycocalyx of biofilm, fully preserved samples, which may provide much insight into the development of *B. subtilis* biofilms.

**Materials and Methods:**

Here, the evaluation of the fixation of ABL technique for the study of *B. subtilis* biofilms was carried out *in situ*, on Congo red agar. In doing so, the comparison to commonly use conventional EDC technique for sample fixation, and observation was carried out. Observations were based on SEM over 30 samples.

**Results:**

Overall, ABL technique provided excellent observation of biofilms formed *in situ*, on Congo red agar, and revealed slime structures, which have not been observed, much in standard EDC fixation or earlier in other studies of these biofilms in *B. subtilis*.

**Conclusion:**

This study reported the appropriate use of ABL in the fixation technique for the preservation of biofilm of *B. subtilis*.

## Introduction

Some of the bacteria are forming biofilm to attach or grow on living and non-living surfaces depending on growth rate and gene transcription [1]. The form of biofilm starts with the beginning of cell division of microcolonies and produces extracellular polymer substances (EPSs. EPSs consist of polysaccharides, and common carbohydrate components in EPS include *D*-glucose, *D*-galactose, *D*-mannose, *L*-fucose, *L*-rhamnose, *D*-glucuronic acid, *D*-galacturonic acid, *L*-guluronic acid, *D*-mannuronic acid, *N*-acetyl- *D*-glucosamine, and *N*-acetyl- *D* -galactosamine [2].

The importance of biofilms is to understand the physiological and ecological characterizations of the microbe by observing exopolysaccharide matrix and evaluation of the mechanical properties of a biofilm. such as heat exchange or diffusion rates of antimicrobials or nutrients through a biofilm by measuring the thickness of it [3]. *B. subtilis* has been used as a model of studying biofilm formation as it is well-known as a Gram-positive bacterium that forms a spore [4,5]. Direct observation [6], scanning electron microscopy (SEM) is a common technique for analysis of biofilm communities in addition to molecular (sequencing or polymerase chain reaction analyses and detection of gene expression) and fluorescent labeling techniques [6,7]. In addition to the survival and forming of *B. subtilis* biofilm, which is dependent on the production of an extracellular matrix, the proper fixation technique is required for the direct observation of the biofilm. The observation of the biofilms of microbes in a cultural medium usually involves fixation of living microbes completely before imaging with SEM. As it is well-known that 97% of the biofilm matrix is water bounds to the capsules of microbial cells [8], therefore, more preservation fixation technique of the biofilm is needed. As Fratesi *et al*. [9] concluded, the different sample fixation techniques give different texture components of the heterogeneous biofilm. Different fixation techniques are well established in the study of Fischer *et al*. [10]. Each of the fixation techniques reveals a different component of the multifaceted biofilms.

Many studies have been tried to investigate the possible protocol of preparation, fixation, or drying of biofilm before SEM observations. For example, hexamethyldisilazane has been used as a solvent for drying biofuels instead of using the critical point drying to avoid impaired cell structure [11], while 2.5% ­glutaraldehyde in phosphate-buffered saline (0.2 M; pH 7.4) has been used to fix biofilm [12]. Conventional N-(3-dimethylaminopropyl)-N’-ethyL-carbodiimide (EDC) fixation technique has been widely used to fix biofilms [13]. However, the sample needs to be preserved in its structure. Alternatively, the alcian blue and lysine (ABL) fixation technique was also used to observe biological samples [14].

However, this EDC fixation technique does not perform well for biofilm sample types, and the effectiveness of SEM in the analysis of biofilms remains to be established. This EDC fixation technique could potentially preserve the structure of biofilms. The present study aimed to evaluate the use of EDC fixation technique and associated SEM for the direct observations and analysis of *B. subtilis* biofilms *in situ* on Congo red agar.

## Materials and Methods

### Ethical approval

The study was approved by the Department of Biological Sciences, University of Babylon, Iraq.

### Study period and location

This study was performed in August 2018 at Department of Biological Sciences, College of Science for Women, University of Babylon.

### Bacterial strains, culture, and media

*B. subtilis* was purchased from Sigma-Aldrich. *B. subtilis* was routinely cultured in Nutrient Broth medium (Fisher Scientific, UK).

### Biofilm formation of B. subtilis on Congo red agar

The formation of biofilm was studied on Congo red agar, which consisted of 15 g of brain-heart infusion broth powder, 20 g of glucose, 50 g of sucrose, 0.8 Congo red dye, 15 g of NaCl, and 20 g of agar. *B. subtilis* was streaked on the Congo red agar and incubated at 37 °C for 7 days. The colors of the former colonies were observed; red color was a negative result, while the black color was a positive result [15].

### Protocol of SEM observations

The observations of the biofilm formation of *B. subtilis* under SEM analyses were included steps of preparing samples before analyses based on the protocol of Erdos [14], Hajibagheri [16], Chavant *et al*. [17] with some modification.

#### Blocks of colonies of B. subtilis formed a biofilm

Using a sterile blade, the blocks of Congo red agar with colonies of *B. subtilis* on the top were cut in 5 mm on each side without disturbing the colony.

#### Primary fixations of the colony blocks

Falcon tube lid was placed in the middle of a glass Petri dish (the inside face upward), and the blocks then were placed around the Falcon lid. After placing the plate in the fume hood in the tissue-lined tray, 0.5 mL of mixed solution (0.25 mL of 4% osmium tetroxide and 0.75 mL cacodylate buffer [100 mM] pH=7.2) was placed into the Falcon tube lid inside of the Petri dish, covered the dish with its lid, wiped with Parafilm, and left it in the tray in the hood for an hour for primary fixation. The blocks were removed from osmium Petri dishes and placed in two wells of a 24-well tissue culture plate to fix either by EDC or ABL techniques.

#### EDC fixation technique

About 3% solution of EDC (3% EDC in 100 mM cacodylate, pH = 7.2) was added to each block without allowing the solution of covering the colonies to wet the agar blocks and left for an hour for primary fixation.

#### ABL fixation technique

The mixture containing 0.63 mL formalin (paraformaldehyde) (16%), 0.50 mL alcian blue (0.75% *w*/*v*), 0.75 mL *L*-lysine hydrochloride (500 mM), and 0.50 mL glutaraldehyde solution (25%) was added to each block without allowing the solution to cover the colonies to wet the agar blocks and left for an hour. Then, either the fixative blocks from EDC or ABL techniques were removed from each well using a clip to a new well. With the same caution, cacodylate buffer (100 mM) was added without allowing the buffer covering the blocks and left for 30 min (this step repeated twice times). Once more, the blocks were returned to the Petri dish and 0.5 mL 1% osmium tetroxide solution was added into the Falcon lid, the plate lid was covered with Parafilm and incubated at constant room temperature for 2 h.

#### Clean the fixed blocks

The blocks were washed twice before dehydration. The blocks were washed with enough ddH_2_O to cover the blocks but not the fixed surface and incubated at constant room temperature for 30 min.

#### Ethanol dehydration of blocks

Ethanol grades (10%, 20%, 40%, 60%, 75%, and 90%) were used for the dehydration of blocks sequentially. The blocks were transferred into the 10% gradient and incubated for 2 h and then sequentially into 20%, 40%, 60%, 75%, and 90%. Then, the blocks were transferred up to 3 times into universal glass bottles containing 5 mL 100% ethanol and incubated for 2 h. Finally, the blocks were transferred carefully into glass Bijou bottles filled with 100% ethanol, and lids were screwed tightly, sealed with Parafilm, and stored at constant room temperature (22 °C).

#### Dehydration of blocks

Liquid CO_2_ was used to dry the blocks by the replacing of the ethanol in the blocks with CO_2_, which would convert the ethanol into a gas without making cell shrug.

#### Sputter coating

The blocks were placed on aluminium cubs with silver glue and coated with gold using a sputter coater.

#### Observations of B. subtilis-formed biofilm under SEM analyzer

SEM observations of *B. subtilis*-formed biofilm, which fixed either by EDC or ABL techniques were performed under SEM analyzer at a voltage of 15 kV.

## Results

### Biofilm formation of B. subtilis

**Figure-1:**
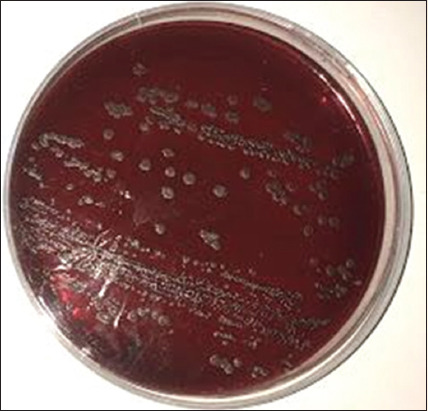
Biofilm formation of *B. subtilis* on Congo red plate after 7 days’ incubation at 37 °C.

*B. subtilis* was grown on Congo red agar plate to confirm the biofilm forming. Figure-1 shows *B. subtilis*-produced black colonies after 7 days of incubation.

### Structural role within the developing B. subtilis biofilm

**Figure-2:**
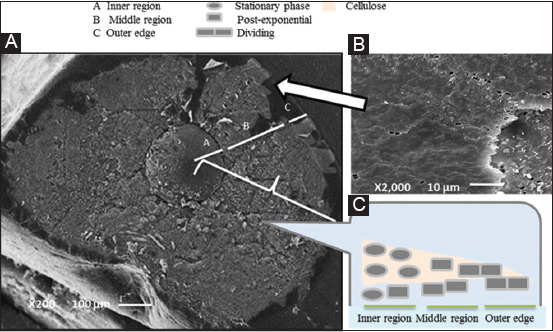
(A) *B. subtilis* biofilm structure. After prolonged incubation, *B. subtilis* forms complex colony type biofilms on agar plates. The colony can be divided into three regions: (A) Inner, (B) middle, and (C) outer. (B) The transfer region between the middle region and the outer edge. (C) Different cell types and their location within the biofilm regions in the cross-section of the colony.

As shown in Figure-2A, the microcolony formed by *B. subtilis* can be divided into three distinct regions: The inner, middle, and outer regions. Furthermore, the proposed cell types and their location within the biofilm regions in the cross-section of the colony are shown in Figure-2C show at the inner and middle regions, stationary and post-exponential, rod-shaped cells are found. In contrast, at the edge zone, lose cells are found Figure-2B.

### Comparison of two fixation techniques for SEM imaging of biofilms

**Figure-3:**
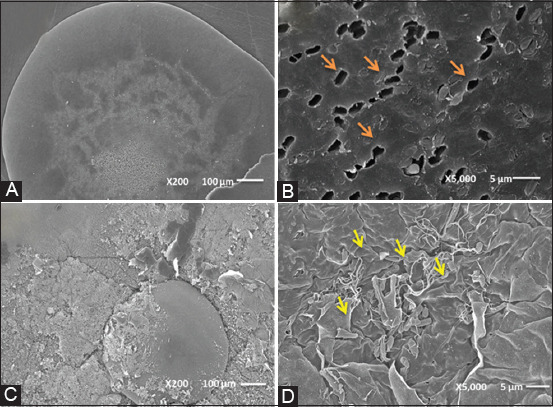
Comparison of biofilm in samples containing *B. subtilis* and prepared for scanning electron microscopy observation using conventional EDC and ABL fixation techniques. (A and B) Images obtained following conventional EDC fixation technique of colony viewing of the coated colony. Orange arrows indicate the section in SEM images where individual cells were lost. (C and D) Images obtained from observation of fixed biofilms with ABL techniques. Yellow arrows indicate slime (extracellular polymers substance) of biofilm and fully preserved biofilm. Images represent an analysis of biofilms fixed in triplicate techniques.

Figure-3 A, C shows the comparison in the fixation technique in the biofilm observation and differences in overall appearance colony and EPS in a sample of *B. subtilis*. Each sample shows rod-shaped bacteria. Bacteria in samples fixed by EDC fixation technique appear to lose their biofilms (Figure-3B, orange arrows), and it is difficult to distinguish the biofilm from its thin slime layer. While bacteria from samples fixed by ABL fixation technique are preserved, and some thin filament similar to a spider’s web as shown in Figure-3D (yellow arrows) and could be related to EPS. The observation of EPS in biofilm is an important finding as Leriche *et al*. [18] stated that the EPS constitutes the higher portion of the biofilm volume and none of the methods can characterize EPS *in situ* like other characterization of EPS in suspended cultures.

## Discussion

*B. subtilis* is capable of forming microcolonies on agar plates that are structurally significant of the biofilms formed by *Escherichia coli* [19] or *Vibrio* *cholerae* [20]. In images from both SEMs, which were fixed by conventional EDC and ABL fixation techniques, the bulk biofilm matrix appeared saturated with the formless sparkling material, which covered individual cells, consistent with previous SEM observations of *B. subtilis* biofilms [21-23]. As shown, the biofilm structure has different features that distinguish between each region. The inoculated cells formed an internal region that corresponds to the initial growth on agar while the middle and outer areas show the appearance of wrinkles consisting of concentric rings leading to a smooth area in the edge region.

The proposed cell types and their location within the biofilm regions in the cross-section of the colony observation were similar to the proposed dividing of the structure of biofilm in *E. coli* [2] with certain ­differences in the curly fibers and cellulose, which genetically reviewed for the molecular mechanisms underpinning the microcolony formation process [24].

In terms of the comparison of two fixation techniques for SEM imaging of biofilms, both techniques provide equal morphological information and an idea of the biofilm forming from SEM observations. However, the main finding is the differences in EPS preservation, which is preserved by the ABL fixation technique and lost by EDC fixation technique. Consequently, these observations confirm that the ABL fixation technique does not lead to a biofilm contraction despite the time used for the entire fixing procedures were equal for both techniques (EDC and ABL).

## Conclusion

In most natural environments, some microorganisms tend to develop biofilms glued together with the slime. Although the research community has investigated the SEM observation of biofilm formation, there is still a need for effective preparation of sample associated biofilm.

Overall, although conventional EDC fixation technique has been used in the observation of biofilm [13], the present study reported the first use of ABL fixation technique in the direct observation of biofilm *in situ*, Congo red agar. The advantages of using ABL fixation technique for SEM observation of biofilm showed the presence of EPS of biofilm not like the observation of biofilm that was fixed by EDC fixation technique, which showed the absence of EPS. A clearer observation of the biofilms in the site should enhance ecological and clinical decision-making and provide the groundwork for advance research on novel control approaches [25].

This study concludes that the ABL fixation technique can be used for the fixation of biofilms for SEM examination. It is an appreciated and cheap alternative to the EDC fixation technique.

## Author’s Contributions

NMTJ performed the study, prepared and analyzed samples by scanning electron microscopy, written the manuscript and critically analyzed the figures and approved the final manuscript.
